# Radiobiological modeling analysis of the optimal fraction scheme in patients with peripheral non-small cell lung cancer undergoing stereotactic body radiotherapy

**DOI:** 10.1038/srep18010

**Published:** 2015-12-11

**Authors:** Bao-Tian Huang, Jia-Yang Lu, Pei-Xian Lin, Jian-Zhou Chen, De-Rui Li, Chuang-Zhen Chen

**Affiliations:** 1 Department of Radiation Oncology, Cancer Hospital of Shantou University Medical College, 7 Raoping Road, Shantou 515031, China; 2 Department of Nosocomial Infection Management, The Second Affiliated Hospital of Shantou University Medical College, 69 North Dongsha Road, Shantou 515041, China

## Abstract

This study aimed to determine the optimal fraction scheme (FS) in patients with small peripheral non-small cell lung cancer (NSCLC) undergoing stereotactic body radiotherapy (SBRT) with the 4 × 12 Gy scheme as the reference. CT simulation data for sixteen patients diagnosed with primary NSCLC or metastatic tumor with a single peripheral lesion ≤3 cm were used in this study. Volumetric modulated arc therapy (VMAT) plans were designed based on ten different FS of 1 × 25 Gy, 1 × 30 Gy, 1 × 34 Gy, 3 × 15 Gy, 3 × 18 Gy, 3 × 20 Gy, 4 × 12 Gy, 5 × 12 Gy, 6 × 10 Gy and 10 × 7 Gy. Five different radiobiological models were employed to predict the tumor control probability (TCP) value. Three other models were utilized to estimate the normal tissue complication probability (NTCP) value to the lung and the modified equivalent uniform dose (mEUD) value to the chest wall (CW). The 1 × 30 Gy regimen is recommended to achieve 4.2% higher TCP and slightly higher NTCP and mEUD values to the lung and CW compared with the 4 × 12 Gy schedule, respectively. This regimen also greatly shortens the treatment duration. However, the 3 × 15 Gy schedule is suggested in patients where the lung-to-tumor volume ratio is small or where the tumor is adjacent to the CW.

Retrospective studies have established that stereotactic body radiotherapy (SBRT) treatment is effective for medically inoperable early-stage non-small cell lung cancer (NSCLC)^1^,^2^,^3^. SBRT can reportedly achieve disease-free survival (DFS), local control (LC) and distant control (DC) outcomes similar to those of surgery with minimal associated toxicity^4^,^5^,^6^.

Although SBRT has been widely used for the treatment of early-stage NSCLC^7^,^8^,^9^,^10^, the optimal fraction scheme (FS) has remained unclear. The FS in the literature varies extensively, from 1×25^11^,^12^,^13^ to 3×20 Gy^8^,^14^,^15^ and the corresponding biologically effective dose (BED) ranges from 87.5 to 180 Gy, using a α/β ratio of 10. Higher dose schedules will increase acute toxicities to normal tissues. Additionally, previous studies have only used overall survival (OS) or LC as endpoints when assessing the dose-response relationship with clinical outcomes; however, these studies failed to consider complications that may arise in normal tissues. Consequently, the optimal FS to maintain tumor control and to minimize toxicity for NSCLC patients requires further investigation.

Radiobiological modeling has the potential to link the dosimetric differences with radiobiological responses; this approach was recently used to predict the possibility of dose escalation for patients with esophageal cancer^16^ and primary prostate cancer^17^.

This study aimed to determine the optimal FS by radiobiologically modeling the tumor control probability (TCP) and normal tissue complication probability (NTCP) values of SBRT treatment of NSCLC, taking the 4×12 Gy scheme as a reference. To exclude the impact of tumor size, we only recruited patients bearing T1 or metastatic (≤3 cm) tumors for this study.

## Methods and Materials

### Ethics statement

The protocol was approved by the Ethics Committee of the Cancer Hospital of Shantou University Medical College. Because this is not a treatment-based study, our institutional review board waived the need for written informed consent from the participants. However, the patient information was kept anonymous to protect their confidentiality. The methods in the study were performed in accordance with the approved guidelines and regulations.

### Patient characteristics and CT scanning

CT simulation data for sixteen patients previously diagnosed with primary or metastatic NSCLC who harbored single peripheral lesions ≤3cm were used in this study. The patient characteristics were presented in Table 1. The median tumor diameter was 2.3 cm, and the median tumor volume was 4.9 cc. The patients were simulated in the supine position with a vacuum bag (Medtec Medical, Inc. Buffalo Grove, IL) or a thermoplastic mask (Guangzhou Klarity Medical & Equipment Co., Ltd, Guangzhou, China) restriction system. Twelve of the sixteen patients underwent respiratory-correlated four-dimensional computed tomography (4DCT) scans using Brilliance CT with Big Bore (Cleveland, OH, USA). For the remaining four patients, respiratory motion was determined on a patient-specific basis in each of the three dimensions while considering respiratory motion, which was visualized with the aid of fluoroscopy due to the patients’ refusal. CT images were obtained at a 3 mm thickness during scanning. The CT images were then transferred to an Eclipse treatment planning system (Version 10.0, Varian Medical System, Inc., Palo Alto, CA) for target delineating, organs at risk (OARs) contouring and treatment planning.

### Delineation of target volume and OARs

For 4DCT images, the internal target volume (ITV) was defined as the combination of the gross tumor volume (GTV) on the ten phases of the 4DCT scan under the CT pulmonary windows. For the 3DCT images, the ITV was expanded according to the tumor motion on fluoroscopy. To account for set-up uncertainties and potential baseline tumor shift, a planning target volume (PTV) was created by adding a uniform 5 mm margin expansion to the ITV. For normal tissue contouring, the whole lung was limited to the air-inflated lung parenchyma, and the GTV and trachea/ipsilateral bronchus were excluded according to the RTOG 0915 report^18^. The chest wall (CW) was segmented from the corrected lung edges with a 2 cm expansion in the lateral, anterior, and posterior directions, excluding the lung volume and the mediastinal soft tissue^19^,^20^,^21^. If the 2 cm expansion extended outside the body, then the contour extended only as far as the external patient surface^20^. To avoid cumbersome contouring of the entire CW, we defined it within a 3 cm limit in the head-to-feet direction from the PTV^19^.

### Treatment planning

Ten different FS of 1 × 25 Gy, 1 × 30 Gy, 1 × 34 Gy, 3 × 15 Gy, 3 × 18 Gy, 3 × 20 Gy, 4 × 12 Gy, 5 × 12 Gy, 6 × 10 Gy and 10 × 7 Gy were prescribed according to previous publications. The treatment was planned using the Eclipse treatment planning system and conducted on the averaged 4DCT. All plans were designed on a TrueBeam LINAC with a 6 MV flattening filter free (FFF) photon beam and a maximum dose rate of 1400 MU/min. Plans were created using dual partial arcs to prevent irradiation of the contralateral lung. The collimator angles for all plans were set to 30° for one arc and 330° for the other arc to minimize the contribution of the tongue-and-groove effect to the dose. Optimization was conducted using the progressive resolution optimizer (PRO_10028) algorithm implemented in Eclipse 10.0. The optimizing objectives were adjusted to ensure that the maximum dose was between 120%–130% of the prescription dose and centered in the GTV. Dose calculation was performed using the anisotropic analytical algorithm (AAA_10028) with a grid resolution of 1 mm while accounting for the heterogeneity correction. The final dose was normalized to ensure that 95% of the PTV received the prescription dose. All prescription dose constraints and critical organ dose-volume limits met the criteria of the RTOG 0915 protocol^18^ and other publications^22^.

### Radiobiological modeling

Both the TCP and NTCP values were calculated using in-house developed programs with MATLAB 7.0 (MathWorks, USA). The TCP was calculated using five different radiobiological models: the Martel model, Fenwick model, Webb-Nahum model, equivalent uniform dose (EUD)-based model and Nitin model. The Webb-Nahum model is a general-type of model with modeling parameters originating from *in vitro* experiments using lung cancer cell lines, whereas the parameters of the other four models are based on the results of clinical trials on lung cancer. Notably, the Nitin model was generated by retrospectively analyzing 504 NSCLC tumors treated with a variety of SBRT schedules^23^. We used the EUD based Lyman-Kutcher-Burman (LKB) model and Fenwick model to estimate the NTCP value to the lung. Radiation-induced CW toxicities were predicted using the modified equivalent uniform dose (mEUD) model with moderate weighting^24^. The specific calculation procedure was as follows: first, the statistics from a cumulative dose volume histogram (cDVH) of the GTV, lung and CW were exported at a resolution of 5 cGy and imported into MATLAB software. Second, the in-house developed program converted the cDVH to the differential DVH (dDVH) according to Gay’s method^25^. Third, the program converted the dose in each volume element to a biologically equivalent dose in 2 Gy fractions (EQD_2_) using the formula reported by other publications^26^,^27^. Finally, the main program automatically calculated the TCP and NTCP results using different radiobiological models. The value of α/β ratio was assigned to 10 Gy for the tumor (an appropriate value for lung tumor) during the EQD_2_ and BED conversion^15^,^28^. α/β values of 1.3 and 3 Gy were assigned to estimate the NTCP to the lung and the mEUD to the CW, respectively^29^,^30^. We used the formula BED_10_ = n×d×[1 + d/(α/β)] to determine the relationship between BED_10_ and dose-response. n and d represent the number of fractions and dose per fraction, respectively, as described by Liu’s work^28^.

The following five TCP predicting models were taken from the literatures:

The Martel model^31^


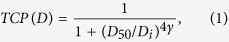


where *D*_*i*_ is the uniform dose irradiated to the fractional volume *V*_*i*_. *D*_*50*_ (the dose needed to achieve a 50% probability of tumor control) = 84.5 Gy and *γ* (the normalized slope of the sigmoid-shaped dose response curve at *D*_*50*_) = 1.5, giving local progression-free survival at 30 months;

The Fenwick model in TCP prediction^32^


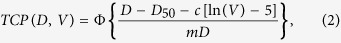


where *D*_*50*_ = 84.6 Gy, *m* = 0.329, *c* = 9.58, *V* is the tumor volume in cm^3^, and Φ is a Gaussian integral;

The Webb-Nahum model^33^


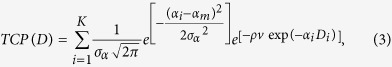


where *α*_*m*_ = 0.30 and *σ*_*α*_ = 0.11, *α*_*m*_ and *σ*_*α*_ are calculated by averaging ten different histological sub-types of human lung cancer cell lines from Carmichael’s report^34^. *ρ* = 10^8^ is the density of clonogenic cells in the tumor^35^, and *v* is the tumor volume in cm^3^;

TCP was calculated for each of the i volume bins *V*_*i*_ of DVH using Equation (1), (2) and (3) and combined using the standard approach.


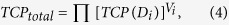


The EUD model^25^


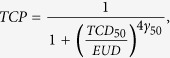



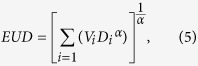


where *TCD*_*50*_(the tumor dose to control 50% of the tumor) = 51.24 Gy and *γ*_*50*_ (the change in TCP expected because of a 1% change in dose about the *TCD*_*50*_) = 0.83 is obtained from Okunieff’s report of a multi-institutional analysis^36^. *α* = 0.30. *D*_*i*_ is the uniform dose irradiated to the fractional volume *V*_*i*_;

The Nitin model^23^


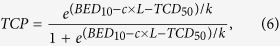


where *BED*_*10*_ is the BED calculated using the linear quadratic (LQ) model with *α/β* = 10 Gy, and *c, TCD*_*50*_, and *k* are 10 Gy/cm, 0 Gy, and 31 Gy, respectively. *L* is the maximal tumor diameter;

The following three NTCP predicting models were also taken from the literatures:

The LKB model^37^


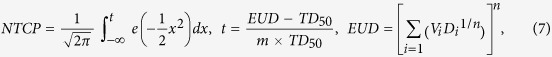


where *n* = 1.00, *m* = 0.45 and *TD*_*50*_ = 26.8^38^. *D*_*i*_ is the uniform dose irradiated to the fractional volume *V*_*i*_;

The Fenwick model in NTCP prediction^32^


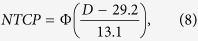


where *D* is mean lung dose, and Φ is the integrated normal distribution;

The mEUD model^24^


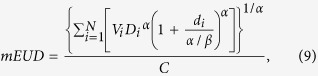


where *N* is the total number of dose bins, *d*_*i*_ is the fractional dose for the subvolume of *V*_*i*_ of the 100 cc high-dose region in the DVH. *D*_*i*_ is total dose of the 100cc dDVH. *α* = 5 (moderate weighting). The numerator C is a constant.

### Determining the optimal FS

As no consistent criterion for evaluating the optimal FS exists, we use the 4 × 12 Gy schedule as a referential FS because it is the most commonly used dose schedule when the tumor is less than 3 cm^39^,^40^,^41^ and extensive publications have demonstrated its safety, efficacy, and minimal toxicity for SBRT treatment of lung cancer^8^,^42^,^43^,^44^. Therefore, the optimal FS in this study is defined as the FS with TCP and NTCP values comparable to the 4 × 12 Gy dose schedule while providing the fewest fractions.

### Statistical analysis

All data in this study are presented as the mean±standard deviation (SD). Differences between plans were assessed by the Wilcoxon signed-rank test in two related samples using SPSS 17.0 (Chicago, IL). Differences were considered significant when *p* < 0.05.

## Results

### Comparison of tumor BED_10_ values for different FS

The calculated tumor BED_10_ (with a α/β ratio of 10) values for 1 × 25 Gy, 1 × 30 Gy, 1 × 34 Gy, 3 × 15 Gy, 3 × 18 Gy, 3 × 20 Gy, 4 × 12 Gy, 5 × 12 Gy, 6 × 10 Gy and 10 × 7 Gy were listed in Table 2. The tumor BED_10_ values, in descending order, were 3 × 20 Gy > 3 × 18 Gy > 1 × 34 Gy > 5 × 12 Gy > 1 × 30 Gy = 6 × 10 Gy > 10 × 7 Gy > 3 × 15 Gy > 4 × 12 Gy > 1 × 25 Gy.

### Effect of the tumor BED_10_ on the TCP and NTCP values

**Figure 1:**
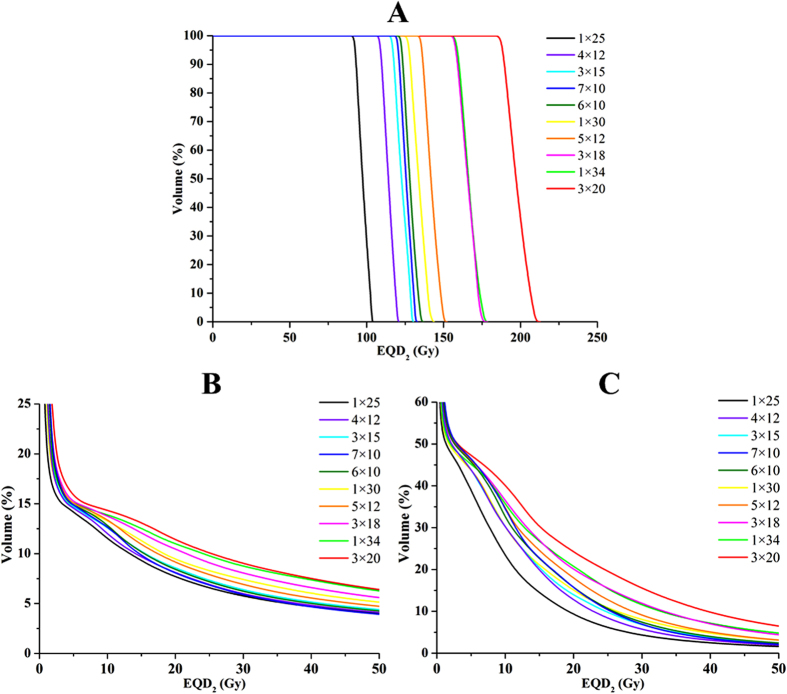
cDVH of the GTV, lung and CW in ten fraction regimens after EQD_2_ conversion. (**A**) cDVH of the GTV. (**B**) cDVH of the lung. (**C**) cDVH of the CW.

The calculated TCP and NTCP values in sixteen patients were also listed in Table 2. The difference in the TCP and NTCP values between either of the two FS was statistically significant (*p* < 0.05). TCP positively correlated with the tumor BED_10_ in five TCP radiobiological models. Additionally, the number of fractions also influenced the NTCP estimation of an equivalent tumor BED_10_. The 3 × 20 Gy scheme provided the highest TCP (98.2% on average), NTCP (13.3% on average for the lung) and mEUD values (179.0 on average for the CW), while the 1 × 25 scheme attained the lowest TCP (82.8% on average), NTCP (4.9% on average for the lung) and mEUD values (90.4 on average for the CW). The cDVH for the GTV, lung and CW from ten regimens after EQD_2_ conversion were presented in Fig. 1. The EQD_2_-based cDVH reflected the difference of EQD_2_ irradiated to the tumor and normal tissues.

### Determination of the optimal FS

When R > 400 or D was between 5.5–15 mm, the 1×30 Gy schedule improved the TCP estimation by 4.2% (Table 2) and significantly reduced the fractionation while maintaining NTCP and mEUD values slightly higher to those for the 4×12 Gy regimen (Table 3, 6.0% vs 4.1% for the NTCP value to the lung and 80.7 vs 62.2 for the mEUD value to the CW). The 1×25 Gy schedule was excluded due to its much lower TCP value (82.8% on average) compared with the 4×12 Gy scheme (89.9% on average).

Conversely, when R ≤ 400 and D ≥ 15 or when D ≤ 5.5 mm, the 3 × 15 Gy scheme was recommended due to the 2.2% higher TCP prediction (Table 2) and only slightly higher NTCP and mEUD values to the lung and CW, respectively (Table 3, 13.4% vs 10.9% for the NTCP value to the lung and 142.9 vs 127.1 for the mEUD value to the CW). All the NTCP and mEUD comparisons between either of the two FS were statistically significant (*p* < 0.05). Although the 6×10 and 10×7 Gy FS predicted even higher TCP values and comparable NTCP and mEUD values, these schemes required more fractionation than the 3×15 Gy scheme and were thus not recommended.

## Discussion

Our analysis of the calculated TCP and NTCP values for lung tumors (≤3cm) using radiobiological modeling suggested that a higher BED_10_ is associated with improved TCP and that the NTCP to the lung and mEUD value to the CW are influenced by both the BED_10_ and number of fractions. We determined the most optimal FS were 1×30 and 3×15 Gy for different tumor locations in patients with peripheral NSCLC whose lesions are ≤3cm. To our knowledge, our study is the first to use radiobiological models to predict the TCP and NTCP values from ten FS for SBRT treatment of lung cancer.

Radiation pneumonitis (RP) and radiation-related CW toxicities are the two most common radiotherapy-induced side effects in patients with NSCLC undergoing SBRT. The incidence of RP and CW toxicities range from 10% to 20.3%^45^,^46^,^47^,^48^ and from 8.3% to 32.8%^21^,^24^,^49^,^50^,^51^,^52^, respectively. A systematic review of 3201 patients with localized stage I NSCLC treated with SBRT revealed 2-year OS and LC values of 70% and 91%^4^, respectively; therefore, the impact of radiation-related complications on the quality of life of these patients warrants further attention. Based on our analysis of the ten dose schedules, we recommend the use of the 1×30 and 3×15 Gy regimens for the treatment of patients with peripheral NSCLC whose tumors are ≤3cm because this strategy results in comparable NTCP values while shortening the treatment duration compared with the 4×12 Gy dose schedule.

Our radiobiological modeling is consistent with the results of previous retrospective analyses of the dose response for lung SBRT using large sample sizes. *(1)* Onishi *et al.* observed that BED_10_ ≥ 100 Gy significantly improved both LC and 3-year OS in a cohort of 245 patients from multiple Japanese institutions^53^, and other studies further confirmed this finding^7^,^8^. The 7.1% increase (4 × 12 Gy vs 1 × 25 Gy) in the absolute value of the TCP for a BED_10_ of 105.6 Gy observed in this study supports the findings of several clinical investigations; however, the improvement predicted in our study was smaller due to the use of different tumor stages and various fraction regimens in other clinical studies. *(2)* Videtic *et al.* reviewed the outcomes of 2 SBRT schedules (30 Gy and 34 Gy) for 80 patients with medically inoperable early-stage lung cancer. Both regimens provided equivalent LC and OS rates^54^. We observed that the 1×34 Gy schedule provided a median improvement of only 3% in the predicted TCP compared with the 1×30 Gy scheme, an improvement too small to be detected in the 80 patients in Videtic’s study. Moreover, the 3.7% absolute increase in toxicities to the lung and the 27.9% relative increase in toxicities to the CW that were predicted in our study for the 1×34 Gy schedule are consistent with the 7.3% and 16.0% of patients who experienced toxicity in response to 30 Gy and 34 Gy in their study, although this difference was not significant. *(3)* A recent clinical study conducted by Li *et al.* reported the use of 70 Gy in 10 fractions to achieve excellent LC while maintaining acceptable toxicities for NSCLC patients^55^. Our analysis also supports Li’s finding: the average TCP in response to the 10×7 Gy regimen was 92.9%, while the NTCP to the lung was only 4.3–5.2% in absolute value and the mEUD value to the CW was the second lowest in the 10 fraction regimens. *(4)* Stephans *et al.* noted that 60 Gy delivered in three fractions attained much higher rates of CW toxicity than 50 Gy delivered in five fractions (18% vs. 4%)^49^. The mEUD model predicted CW-related complications of 179.0 and 103.7 on average for the 3×20 Gy and 6×10 Gy schedules in our study, respectively, indicating an up to 72.6% relative reduction in CW-related complications for multiple fraction regimens. The 5 × 10 Gy scheme in Stephans’s research will likely further reduce the rate of complications. Other studies have also reported the tendency that increasing the number of fractions tended to lower the risk of CW pain (CWP)^21^,^24^,^50^,^51^. Our results are consistent with the results of the above-mentioned clinical studies, indicating that SBRT treatment for patients with NSCLC can be predicted using our models.

Although a calculated BED_10_ ≥ 100 Gy is generally associated with improved outcomes, the benefit and need for BED_10_ values higher than 120 Gy or 150 Gy have not yet been determined in small tumors (≤3 cm)^10^,^15^. Our results for patients with small tumors (≤3 cm) demonstrated that a BED_10_ of 120–130 Gy was sufficient to achieve a TCP of 92.9–95.2% while maintaining acceptable radiation-related toxicities to the lung and CW. Higher BED_10_ values (150–180 Gy) further improved the TCP (97.0–98.2%) but increased the NTCP to the lung by up to 9% in absolute value and increased the risk of radiation-related CW toxicities by up to 43% in relative value.

Radiobiological models can be used to calculate TCP and NTCP values with a focus on the dosimetric difference among different fraction regimens without involving unmeasured and potential confounders, such as pathological sub-type, age, sex, and other radiation therapy uncertainties, such as set-up error and inter- or intrafractional tumor motion that will influence the outcome of the clinical treatment. We believe the proposed method facilitated the comparison of the dose responses of different radiotherapy fraction regimens. However, the application of radiobiological models is somewhat controversial, particularly for hypofractionated radiation therapy. Park *et al.* demonstrated that the universal survival curve (USC) better approximated the experimentally measured survival curves in the ablative, high-dose range than the LQ model beyond the threshold of 6.2 Gy per fraction^56^. However, no publication has shown that the USC can predict clinical datasets for SBRT better than standard models up till now, and the utility of a purely empirical model with an additional adjustable parameter for predicting *in vitro* data is questionable. Conversely, accumulating clinical evidence has confirmed the accuracy of LQ-based TCP and BED models. Guckenberger *et al.* suggested that the traditional LQ formalism accurately modeled for patients with stage I NSCLC undergoing SBRT based on 395 patients from 13 German and Austrian centers^57^. Shuryak *et al.* also found that LQ-based TCP and BED models can provide significantly better fits to local control data for NSCLC than TCP models using other high-dose models^58^. According to the clinical evidence, we believe that the data acquired in our study utilizing the LQ model are reliable.

Our study has several limitations. *(1)* The TCP and NTCP values in the study were predicted by radiobiological models, without considering the repopulation and reoxygenation of the tumor cells during the treatment course, and may not reflect actual clinical tumor control and normal tissue complications. However, we assume that the proposed method feasibly predicts the radiobiological response, because the outcomes of this study are consistent with other retrospective SBRT studies of lung cancer. Furthermore, different radiobiological models predicted similar trends, irrespective of the TCP or NTCP prediction. *(2)* We did not consider radiation-induced rib fracture, which is one of the common complications of lung SBRT. Although several reports concluded that the tumor-chest wall distance is a risk factor for this complication^59^, no radiobiological model has been proposed to predict the incidence of rib fracture; thus, we could not easily evaluate rib fracture during the modeling.

## Conclusions

Different radiobiological models yield consistent predictions of TCP and NTCP values for different FS. Higher BED schemes improve the TCP and increasing the number of fractions beneficially reduces the NTCP and mEUD values. The 1 × 30 Gy regimen is preferred to achieve 4.2% higher TCP value and slightly higher NTCP and mEUD values to the lung and CW, respectively, while shortening the treatment duration compared with the 4 × 12 Gy schedule for patients with peripheral NSCLC tumors ≤3 cm. However, the 3 × 15 Gy regimen is recommended in certain patients where the lung-to-tumor volume ratio is small or where the tumor is adjacent to the CW.

## Additional Information

**How to cite this article**: Huang, B.-T. *et al.* Radiobiological modeling analysis of the optimal fraction scheme in patients with peripheral non-small cell lung cancer undergoing stereotactic body radiotherapy. *Sci. Rep.* **5**, 18010; doi: 10.1038/srep18010 (2015).

## Figures and Tables

**Table 1:** Characteristics of sixteen patients with NSCLC undergoing SBRT in order of increasing GTV size.

**Patient**	**Gender**	**Age**	Stage*	**GTV (cc)**
1	F	57	BC metastatic	0.9
2	F	35	NPC metastatic	1.0
3	F	55	T1	2.1
4	M	71	T1	3.1
5	M	64	T1	3.3
6	M	62	T1	3.4
7	M	68	T1	3.6
8	F	59	T1	4.0
9	M	68	T1	4.2
10	F	76	T1	4.2
11	F	63	T1	4.6
12	F	72	T1	5.4
13	F	71	T1	6.9
14	M	62	T1	9.7
15	F	70	T1	10.3
16	M	70	T1	11.6

*Abbreviations:* GTV = gross target volume; BC = breast cancer; NPC = nasopharyngeal carcinoma. Note: *According to the American Joint Committee on Cancer (AJCC), 7th edition.

**Table 2:** Comparison of BED_10_, TCP, NTCP and mEUD values in different FS.

**Parameterr**	**1 × 25 Gy**	**4 × 12 Gy**	**3 × 15 Gy**	**10 × 7 Gy**	**6 × 10 Gy**
BED_10_	87.5	105.6	112.5	119	120
TCP					
Mar (%)	68.6 ± 4.6	85.0 ± 2.5	89.7 ± 2.0	91.1 ± 1.4	92.1 ± 1.4
Fen (%)*	88.7 ± 3.3	92.1 ± 2.2	93.2 ± 1.8	93.5 ± 1.7	93.8 ± 1.6
WN (%)	78.6 ± 2.7	85.9 ± 1.7	88.3 ± 1.4	89.0 ± 1.2	89.6 ± 1.2
EUD (%)	89.1 ± 1.1	93.3 ± 0.6	94.6 ± 0.6	95.1 ± 0.4	95.4 ± 0.4
Nitin (%)	88.8 ± 1.8	93.4 ± 1.1	94.7 ± 0.9	95.6 ± 0.8	95.8 ± 0.7
Median (%)	82.8 ± 8.7	89.9 ± 4.1	92.1 ± 3.0	92.9 ± 2.8	93.3 ± 2.7
NTCP					
Lung					
LKB (%)	5.3 ± 2.8	5.4 ± 2.9	6.3 ± 3.7	5.2 ± 2.6	5.9 ± 3.3
Fen (%)†	4.5 ± 2.4	4.6 ± 2.4	5.3 ± 3.1	4.3 ± 2.2	5.0 ± 2.8
Median (%)	4.9 ± 2.6	5.0 ± 2.7	5.8 ± 3.4	4.7 ± 2.4	5.5 ± 3.0
CW					
mEUD	90.4 ± 41.5	94.7 ± 41.6	106.0 ± 47.1	95.0 ± 38.6	103.7 ± 44.1
**Parameterr**	**1 × 30 Gy**	**5 × 12 Gy**	**1 × 34 Gy**	**3 × 18 Gy**	**3 × 20 Gy**
BED_10_	120	132	149.6	151.2	180
TCP					
Mar (%)	93.7 ± 1.4	95.5 ± 0.9	98.3 ± 0.4	98.1 ± 0.4	99.4 ± 0.2
Fen (%)*	94.3 ± 1.5	95.0 ± 1.3	96.2 ± 0.8	96.2 ± 0.9	97.1 ± 0.6
WN (%)	90.6 ± 1.1	91.9 ± 0.9	94.5 ± 0.6	94.3 ± 0.6	96.1 ± 0.4
EUD (%)	96.0 ± 0.5	96.7 ± 0.3	98.0 ± 0.2	98.0 ± 0.2	98.9 ± 0.1
Nitin (%)	95.8 ± 0.7	97.1 ± 0.5	98.3 ± 0.3	98.4 ± 0.3	99.4 ± 0.1
Median (%)	94.1 ± 2.2	95.2 ± 2.0	97.1 ± 1.6	97.0 ± 1.6	98.2 ± 1.4
NTCP					
Lung					
LKB (%)	8.4 ± 5.9	7.2 ± 4.5	12.4 ± 9.8	10.3 ± 7.7	14.3 ± 11.6
Fen (%)†	7.1 ± 5.0	6.1 ± 3.8	10.5 ± 8.3	8.7 ± 6.5	12.2 ± 9.9
Median (%)	7.8 ± 5.4	6.6 ± 4.2	11.5 ± 9.0	9.5 ± 7.0	13.3 ± 10.7
CW					
mEUD	127.1 ± 59.5	118.7 ± 51.5	162.6 ± 76.4	148.2 ± 66.6	179.0 ± 81.0

*Abbreviations:* BED_10_ = biologically effective dose (with a α/β ratio of 10); CW = chest wall; TCP = tumor control probability; NTCP = normal tissue complication probability; Mar = Martel model; Fen = Fenwick model; WN = Webb-Nahum model; EUD = equivalent uniform dose model; Nitin = Nitin model; LKB = Lyman-Kutcher-Burman (LKB) model; mEUD = modified equivalent uniform dose model. Statistical significance (*p* < 0.05) was found between either of the two groups. ^*^Indicates the Fenwick model for TCP prediction; ^†^Indicates the Fenwick model for NTCP estimation to the lung.

**Table 3:** Impact of tumor location on the NTCP and mEUD values to the lung and CW in different FS.

**Factor**	**1** × **25 Gy**	**4** × **12 Gy**	**3** × **15 Gy**	**10** × **7 Gy**	**6** × **10 Gy**
R, D (mm)					
R≤400 and D≥15	10.7 ± 1.2*	10.9 ± 1.3*	13.4 ± 1.5*	10.0 ± 1.2*	12.2 ± 1.4*
R > 400 or D < 15	4.0 ± 0.5*	4.1 ± 0.5*	4.7 ± 0.6*	4.0 ± 0.5*	4.5 ± 0.5*
D (mm)					
≤5.5	123.0 ± 27.2†	127.1 ± 27.6†	142.9 ± 30.9†	125.0 ± 25.7†	138.0 ± 29.2†
>5.5	57.8 ± 22.9†	62.2 ± 23.0†	69.0 ± 26.0†	65.1 ± 22.0†	69.3 ± 24.8†
**Factor**	**1 × 30 Gy**	**5 × 12 Gy**	**1 × 34 Gy**	**3 × 18 Gy**	**3 × 20 Gy**
R, D (mm)					
R≤400 and D≥15	20.0 ± 2.3*	16.0 ± 1.8*	32.1 ± 3.7*	25.6 ± 3.0*	37.8 ± 4.3*
R > 400 or D < 15	6.0 ± 0.7*	5.3 ± 0.6*	8.5 ± 1.0*	7.2 ± 0.8*	9.7 ± 1.1*
D (mm)					
≤5.5	173.5 ± 39.4†	158.8 ± 34.0†	222.1 ± 49.8†	200.4 ± 43.6†	242.2 ± 53.2†
>5.5	80.7 ± 33.3†	78.6 ± 29.0†	103.2 ± 44.2†	96.0 ± 37.2†	115.7 ± 45.5†

Abbreviations: R = lung-to-tumor volume ratio; D = tumor-to-CW distance. Statistical significance (*p* < 0.05) was found between either of the two groups.

^*^Note: NTCP value to the lung.

^†^mEUD value to the chest wall.
